# CB1 Receptor Activation on VgluT2-Expressing Glutamatergic Neurons Underlies Δ^9^-Tetrahydrocannabinol (Δ^9^-THC)-Induced Aversive Effects in Mice

**DOI:** 10.1038/s41598-017-12399-z

**Published:** 2017-09-26

**Authors:** Xiao Han, Yi He, Guo-Hua Bi, Hai-Ying Zhang, Rui Song, Qing-Rong Liu, Josephine M. Egan, Eliot L. Gardner, Jing Li, Zheng-Xiong Xi

**Affiliations:** 1 Molecular Targets and Medications Discovery Branch, Intramural Research Program, National Institute on Drug Abuse, Baltimore, MD 21224 USA; 2 Beijing Institute of Pharmacology and Toxicology, Beijing, 100850 China; 3 Laboratory of Clinical Investigation, Intramural Research Program, National Institute on Aging, Baltimore, MD 21224 USA

## Abstract

Cannabis can be rewarding or aversive. Cannabis reward is believed to be mediated by activation of cannabinoid CB1 receptors (CB1Rs) on GABAergic neurons that disinhibit dopaminergic neurons in the ventral tegmental area (VTA). However, little is known about the mechanisms underlying cannabis aversion in rodents. In the present study, CB1Rs are found not only on VTA GABAergic neurons, but also on VTA glutamatergic neurons that express vesicular glutamate transporter 2 (VgluT2). We then used Cre-Loxp transgenic technology to selectively delete CB1Rs in VgluT2-expressing glutamatergic neurons (VgluT2-*CB1* ^−/−^) and Cre-dependent viral vector to express light-sensitive channelrhodopsin-2 into VTA glutamatergic neurons. We found that photoactivation of VTA glutamatergic neurons produced robust intracranial self-stimulation (ICSS) behavior, which was dose-dependently blocked by DA receptor antagonists, but enhanced by cocaine. In contrast, Δ^9^-tetrahydrocannabinol (Δ^9^-THC), the major psychoactive component of cannabis, produced dose-dependent conditioned place aversion and a reduction in the above optical ICSS in VgluT2-cre control mice, but not in VgluT2-*CB1* ^−/−^ mice. These findings suggest that activation of CB1Rs in VgluT2-expressing glutamate neurons produces aversive effects that might explain why cannabinoid is not rewarding in rodents and might also account for individual differences in the hedonic effects of cannabis in humans.

## Introduction

Humans have used marijuana (*cannabis sativa*) for thousands of years for medical and recreational purposes. With on-going legalization of cannabis in the USA, its consumption is now dramatically increasing^1^. Cannabis use and abuse is thought to be associated with its psychostimulant, euphoric and relaxing effects^2,3^. However, not all users enjoy cannabis and some experience dysphoria, anxiety, and depression after its use^4,5^. Cannabis may also produce euphoria, pleasure, or relaxation at one time but depression, fear, or anxiety at another^6,7^. Although these effects have been known for some time, the mechanism underlying these biological effects was not known until the 1970s when Δ^9^-tetrahydrocannabinol (Δ^9^-THC) was identified as the primary psychoactive ingredient of cannabis^8^, and until the 1990s when cannabinoid CB1 and CB2 receptors were identified as the major targets^9,10^. It is now generally accepted that the psychoactive effects of cannabis are mediated through CB1Rs in the brain^11^, and its euphorigenic effects are mediated by Δ^9^-THC-induced activation of the mesolimbic dopamine (DA) system^2,3,12^, which originates in the ventral tegmental area (VTA) in the midbrain and projects mainly to the nucleus accumbens (NAc) and prefrontal cortex (PFC) in the forebrain^12^.

Electrophysiological and anatomic evidence demonstrates that VTA DA neurons receive inhibitory GABAergic and excitatory glutamatergic input^13–15^. Activation of CB1Rs on VTA GABAergic neurons or ﻿GABAergic﻿ afferent terminals leads to a reduction in GABA release and DA neuron disinhibition (or activation)^12,16^, suggesting that cannabis reward might be mediated by activation of CB1Rs on VTA GABAergic neurons^2,3^. This is supported by findings that Δ^9^-THC increases DA release in the NAc as assessed by *in vivo* microdialysis in rats^17,18^ or PET imaging studies in humans^19,20^, although other studies were not able to show this increase in DA^21^. Δ^9^-THC is self-administered by squirrel monkeys^22,23^, showing that it has rewarding effects, but it is not self-administered in rodents^24,25^ or rhesus monkeys^26,27^.

In addition, electrophysiological evidence demonstrates that functional CB1Rs are also expressed in VTA glutamatergic terminals^28,29^. Retrograde release of endocannabinoids can decrease VTA DA neuron activity by inhibiting glutamatergic input^12,30,31^, suggesting that cannabis might produce aversion by activating CB1R in VTA glutamatergic neurons or glutamatergic afferent terminals. However, there is no anatomic evidence indicating whether CB1R is expressed in VTA glutamatergic neurons. Strikingly, in rodents, cannabinoid agonists are either not rewarding or produce overt aversive effects that overshadow any rewarding effects^24,25,32^. The mechanisms underlying such effects are poorly understood. Given that high densities of local glutamatergic neurons are found in the VTA^33^, where they form functional synapses onto VTA dopaminergic neurons^34^, we hypothesized that CB1Rs may also be expressed in local glutamatergic neurons, and activation of CB1Rs in VTA glutamatergic neurons or glutamatergic  afferent terminals may mediate the aversive effects of cannabis.

In the present study, we used both the Cre-Loxp recombination and optogenetic techniques to test this glutamatergic CB1R hypothesis. In this pursuit, we first created CB1-floxed mice and then generated conditional CB1-knockout mice (VgluT2-CB1^−/−^) in glutamatergic neurons that express vesicular glutamate transporter 2 (VgluT2). We then used immunohistochemistry and RNAscope *in situ* hybridization assays to examine whether CB1Rs are expressed in VTA GABAergic neurons﻿ and glutamatergic neurons. Next, conditioned place preference and intracranial self-stimulation (ICSS) maintained by optical activation of VTA glutamatergic neurons were employed to evaluate the effects of Δ^9^-THC on brain reward function. We found that activation of CB1Rs in VTA glutamatergic neurons by Δ^9^-THC produced dose-dependent conditioned place aversion and a significant reduction in optical ICSS, suggesting a cell type-specific CB1R-mediated mechanism underlying Δ^9^-THC-induced aversion in mice.

## Results

### CB1R-immunostaining in the VTA

We first used immunohistochemical assays (IHC) to detect CB1R expression in the VTA-containing midbrain. Figure S1-A shows CB1- and TH-immunostaining, illustrating that CB1Rs (green) are highly expressed in the substantia nigra pars reticulata (SNr), not in the VTA or substantia nigra pars compacta (SNc). However, under  high magnification, CB1-immunostaining was found in the VTA﻿ and SNc, but mainly in cell membranes and nerve fibers, not in cell bodies of neurons (Fig. S1-B). Such patterns of CB1R-staining prevented the further use of IHC to dissect the phenotype(s) of neurons that express CB1Rs since nerve fibers from different types of neurons were intertwined.

### Generation of VgluT2-*CB1*^*‒/‒*^ mice

To dissect the role of CB1Rs in different phenotypes of VTA neurons in cannabis reward and aversion, we first created CB1^flox/flox^ mice in order to generate conditional CB1R-knockout (*CB1* ^‒/‒^) mice in glutamatergic neurons that express VgluT2 (i.e., VgluT2-*CB1* ^‒/‒^ mice). Figure S2-A shows the CB1 allele in wild-type mice. Figure S2-B shows CB1-floxed-neo allele in the heterozygous CB1^flox/wt^ mice to generate CB1^flox/flox^ mice (Fig. S2-C)^35^. VgluT2-*CB1* ^‒/‒^ mice were obtained by crossing CB1^flox/flox^ mice with VgluT2-Cre mice in which Cre recombinase is expressed under VgluT2 promotors. The homozygous VgluT2-*CB1* ^*‒/‒*^ mice and their wildtype (WT) control VgluT2-Cre littermates (VgluT2-﻿*CB1﻿* ^+/+^) were viable and fertile. No significant differences in general health conditions (feeding, locomotion, body weight) were observed between VgluT2-*CB1* ^−/−^ mice and VgluT2-Cre mice.

### CB1 mRNA RNAscope ISH assays

We then used RNAscope, a highly sensitive and specific *in situ* hybridization assay^36^, to examine CB1 mRNA expression in cell bodies of VTA neurons. There are two major subtypes of glutamatergic neurons in the brain – cortical glutamatergic neurons that express VgluT1 and subcortical glutamatergic neurons that express VgluT2^37^. Figure S3-A shows representative images (under low magnification) of VgluT2 mRNA (red) and CB1 mRNA (green) in the brain at the midbrain level, illustrating that VgluT2 is mainly expressed in subcortical regions including the red nucleus (RN) and VTA, while CB1 mRNA is mainly expressed in the hippocampus and cerebral cortex in control VgluT2-Cre mice (also see Fig. S3-B). However, compared with that seen in the control mice (Fig. S3-B), CB1 mRNA expression in VgluT2-*CB1* ^−/−^ mice was reduced significantly in both the cortical (including hippocampus) and subcortical regions, particularly in subcortical areas where CB1 mRNA was abolished (Fig. S3-C).

**Figure 1:**
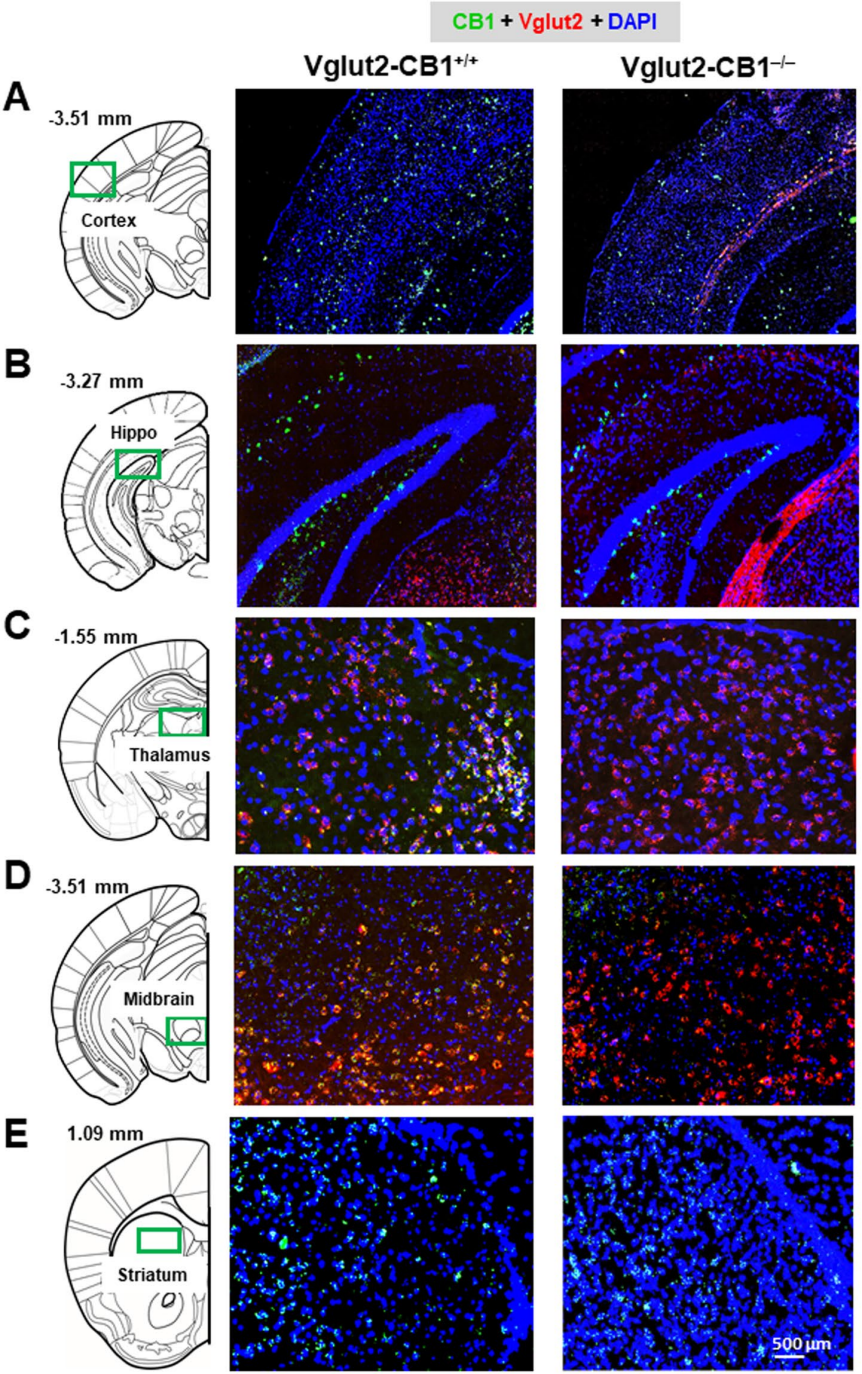
CB1 mRNA expression (by RNAscope) in different brain regions in WT (VgluT2-*CB1* ^+/+^) and VgluT2-*CB1* ^−/−^ mice. Conditional CB1R deletion in VgluT2-*CB1* ^−/−^ mice decreased CB1 mRNA expression (green) in cerebral cortex (**A**) and hippocampus (**B**), while abolished CB1 mRNA expression in subcortical regions such as thalamus (**C**) and midbrain (**D**), compared to their WT littermates. There is no significant difference in CB1 mRNA expression in striatum between the two strains of mice (**E**).

Figure 1 shows CB1 mRNA expression in different brain regions under higher magnification, illustrating that CB1 mRNA levels were decreased in the cortex (Fig. 1A) and hippocampus (Fig. 1B), but absent in the subcortical areas such as thalamus (Fig. 1C) and midbrain (Fig. 1D) in VgluT2-*CB1* ^−/−^ mice. There was no significant change in CB1 mRNA expression in the striatum between the two mouse strains (Fig. 1E). These findings are consistent with VgluT2 distributions in the brain^37^, indicating that the selective CB1R deletion occurs only in VgluT2-expressing glutamatergic neurons in mice.

### CB1 mRNA is expressed in VTA GABAergic and glutamatergic neurons

**Figure 2:**
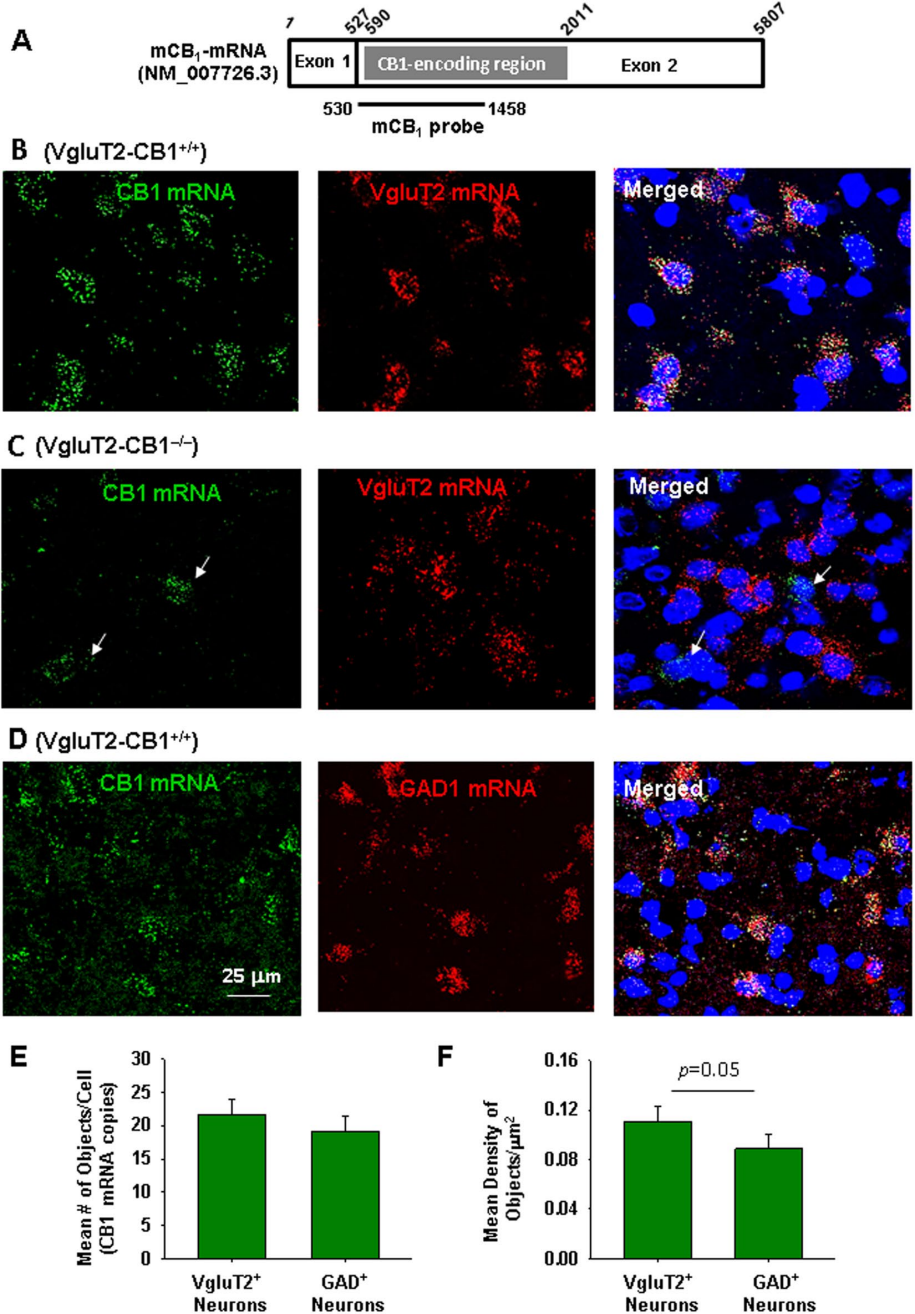
Cellular distributions of CB1 mRNA (by RNAscope) in the VTA. (**A**) Mouse CB1 mRNA structure and the gene target region of the mCB1 probe used in the present study. (**B**,**C)** CB1 mRNA-staining (green) was detected in VTA VgluT2 + glutamatergic neurons (red) in wildtype littermates (**B**), but not VgluT2-*CB1* ^−/−^ mice (**C**). In contrast, CB1 mRNA was detected in non-VgluT2^+^ neurons (white arrows) of VgluT2-*CB1* ^−/−^ mice. (**D**) CB1 mRNA was also detected in VTA GAD1^+^ GABAergic neurons of VgluT2-het control mice. (**E**,**F)** Mean numbers of the detected objects (i.e., CB1 mRNA copies) per cell (**E**) and the mean densities of CB1 mRNA signaling per cell (**F**) in VgluT2^+^glutamatergic neurons (n = 82 cells in 3 mice) and GAD1^+^ GABAergic cells (n = 82 in 3 mice). There is no significant difference in detected objects (**E**), but a marginally significant difference (*p* = 0.05) in CB1 mRNA densities (**F**) between VTA glutamatergic neurons and GABAergic neurons.

A double-staining RNAscope technique was used to examine the cellular distribution of CB1 mRNA in the VTA. Figure 2A shows the mouse CB1 mRNA structure and the target gene region of the CB1 probe used to detect CB1 mRNA in the present study. Figure 2(B,C) shows representative RNAscope *in situ* hybridization (ISH) results, illustrating that CB1 mRNA (green) was detected in almost all VgluT2-positive glutamatergic neurons (red) in the VTA of VgluT2-Het littermates (Fig. 2B), but not in the VTA of VgluT2-CB1^‒/‒^ mice (Fig. 2C). Figure 2D shows that low-to-moderate densities of CB1 mRNA were also detected in VTA GABAergic neurons that express glutamic acid decarboxylase 1 (GAD1). Figure 2(E,F) shows mean CB1 mRNA levels in both VTA VgluT2^+^ glutamatergic and GAD1^+^ GABAergic neurons, illustrating no significant difference in staining objects per cell (Fig. 2E, t = 0.79, *p* > 0.05), but a marginally significant difference in staining density per cell (Fig. 2F, t = 1.38, *p* = 0.05), suggesting that CB1 mRNA level is higher in VTA glutamatergic neurons than in VTA GABAergic neurons.

### Δ^9^-THC produces conditioned place aversion in mice

**Figure 3:**
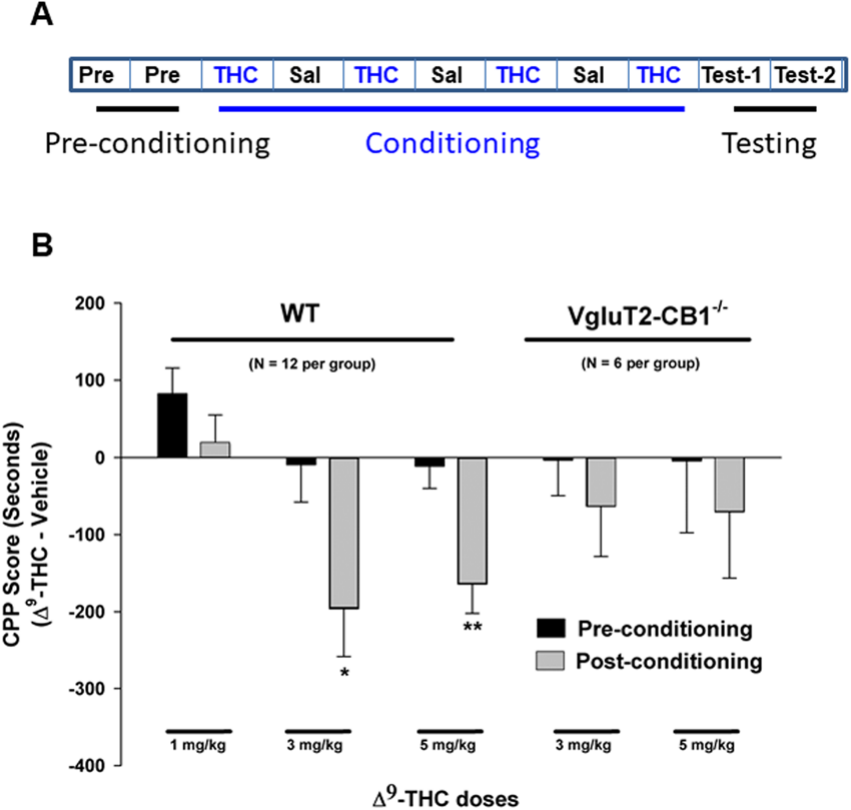
Δ^9^-THC-induced condition place aversion in mice. (**A**) Schematic diagram illustrating the procedure for Δ^9^-THC-induced conditioned place preference (CPP) or aversion (CPA). (**B**) Δ^9^-THC produced a significant dose-dependent CPA in WT littermates, an effect that was significantly attenuated in VgluT2-*CB1* ^−/−^ Mice. *p < 0.05, **p < 0.01, compared to preconditioning in each dose group.

We then used the conditioned place preference (CPP) or aversion (CPA) procedures to compare the rewarding or aversive effects of Δ^9^-THC between VgluT2-*CB1* ^*‒/‒*^ mice and their wildtype control littermates. Figure 3A shows the general experimental procedure. Systemic administration of Δ^9^-THC (1, 3, 5 mg/kg, i.p.) produced dose-dependent conditioned place aversion in wildtype littermates (Fig. 3B, left panels). However, this effect was significantly attenuated in VgluT2-*CB1* ^*‒/‒*^ mice (Fig. 3B, right panels).

### Photoactivation of VTA glutamatergic neurons is rewarding

**Figure 4:**
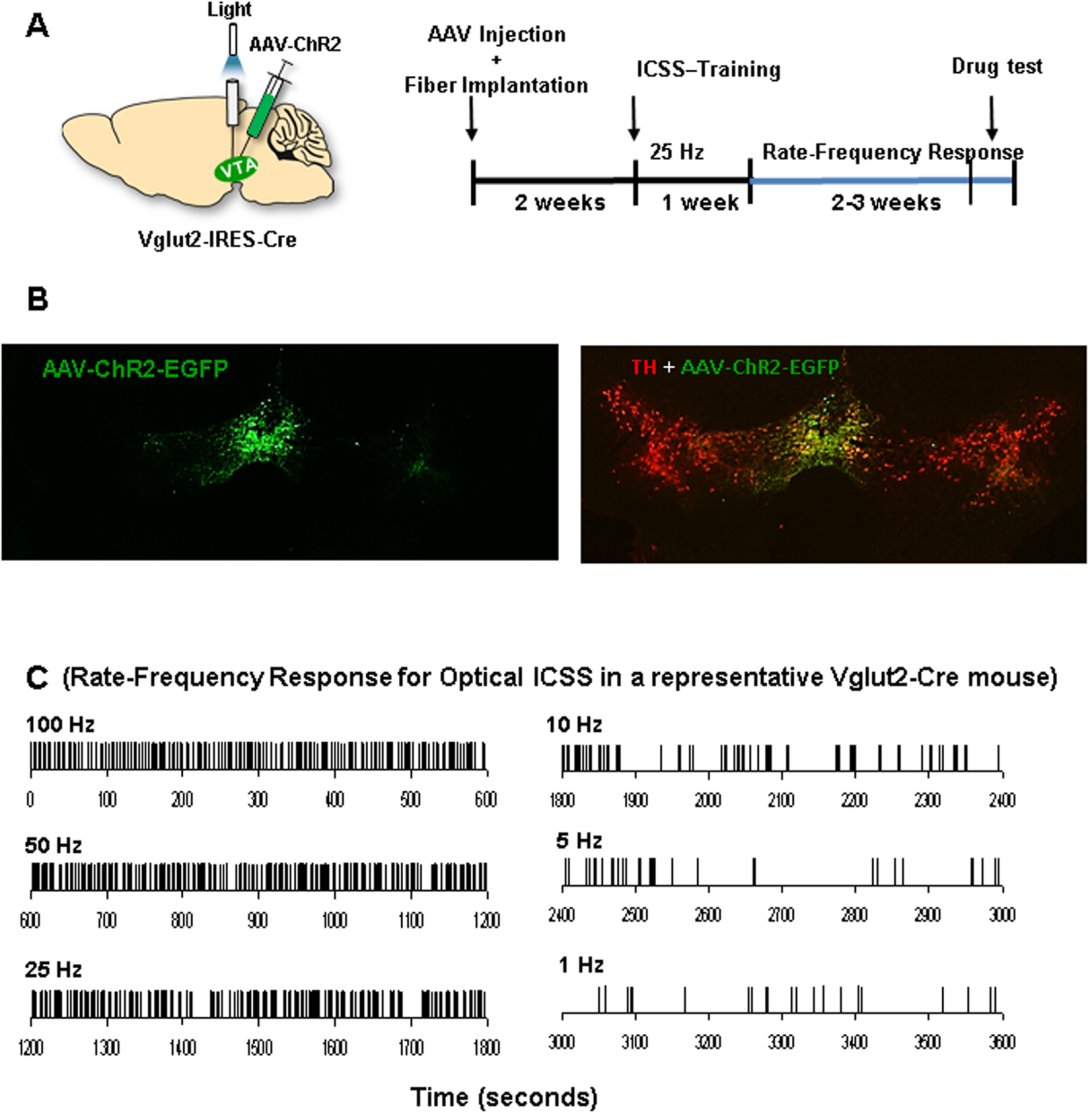
Optical intracranial self-stimulation (oICSS) experiment. (**A**) Schematic diagrams illustrating the target brain region (VTA) of the AAV-ChR2-GFP microinjection and intracranial optical fiber implantation (left), and the time course of the oICSS experiment (right). (**B**) Representative images of AAV-ChR2-EGFP expression in the medial VTA. (**C**) Representative oICSS records in a single session from a single mouse under descending stimulation frequency (from high to low, 10 min per frequency), indicating that photoactivation of VTA glutamatergic neurons in Vglu2-Cre mice induced robust oICSS behavior (lever presses) in a stimulation frequency-dependent manner.

To confirm the above behavioral finding, we next observed the effects of Δ^9^-THC on optical brain-stimulation reward in both genotypes  of mice. Since electrical stimulation used in classical intracranial self-stimulation (ICSS) non-specifically activates all neurons and/or fibers in the target area, electrical ICSS cannot be used to study such cell type-specific mechanisms underlying cannabis reward or  aversion. Therefore, we established a new animal model of optical ICSS (oICSS) maintained by optical activation of VTA glutamatergic neurons in VgluT2-Cre mice. Figure 4A shows the experimental procedures and the time course of the oICSS experiments, illustrating that the AAV-DIO-ChR2-EGFP vector that expresses light-sensitive channelrhodopsin 2 (ChR2) and fluorescent EGFP was microinjected into the VTA in VgluT2-Cre mice, and then an optical fiber was implanted into the VTA ipsilaterally. Figure 4B shows a representative immunostaining image of EGFP and TH, illustrating VgluT2 promoter-driven ChR2-EGFP expression within the medial VTA. Figure 4C shows representative active lever responses observed within a session from a single animal under different stimulation frequencies (from high to low). Response-contingent photoactivation of VTA glutamatergic neurons induced robust active lever presses in a stimulation frequency-dependent manner – the higher the stimulation frequency, the more the active lever presses, and *vice versa*. These findings suggest that photostimulation of VTA glutamatergic neurons is rewarding.

### oICSS: A new animal model for studying brain reward function

**Figure 5:**
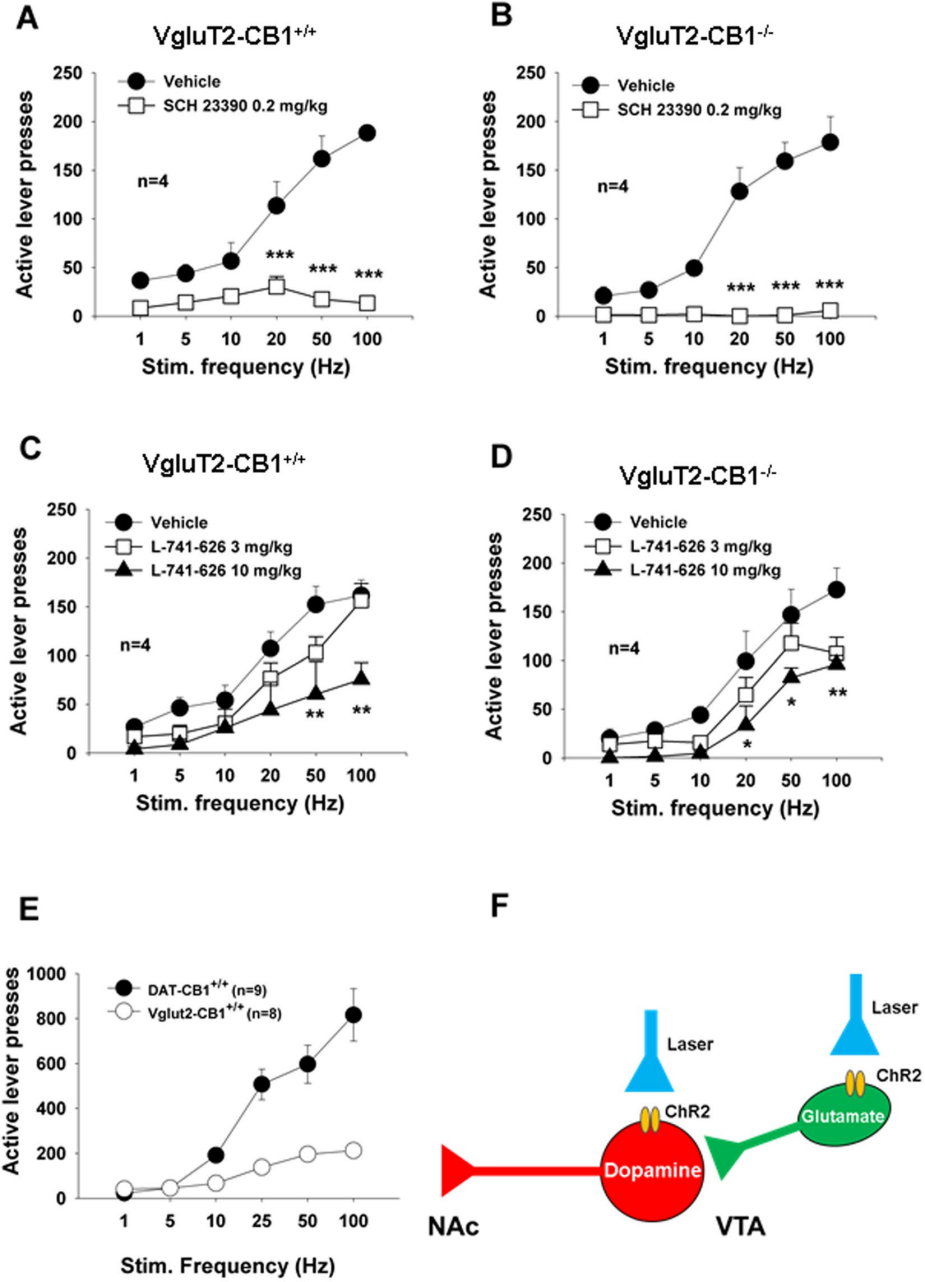
Dopamine-dependent oICSS observed in the present study. (**A/B**) Rate-frequency function curves of oICSS maintained by photoactivation of VTA glutamatergic neurons, indicating that conditional deletion of CB1Rs in VgluT2^+^  glutamatergic neurons (in VgluT2-*CB1* ^−/−^ mice) did not significantly alter oICSS. However, pretreatment with SCH23390, a selective D1 receptor antagonist (0.2 mg/kg, i.p., 15 min prior to testing), significantly inhibited the oICSS in both WT and VgluT2-*CB1* ^−/−^ mice. (**C/D)** Pretreatment with L-741,626, a selective D2 receptor antagonist (3, 10 mg/kg, i.p., 15 min prior to testing), also dose-dependently inhibited the oICSS in both WT and VgluT2-*CB1* ^−/−^ mice. (**E**) Comparison of the oICSS maintained by stimulation of VTA dopaminergic neurons in DAT-Cre mice and VTA glutamatergic neurons in VgluT2-Cre mice, indicating that activation of VTA DA neurons is more potent and effective than activation of VTA glutamatergic neurons in inducing ICSS behavior. (**F**) A schematic diagram illustrating the postulated circuitry underlying these effects.

**Figure 6:**
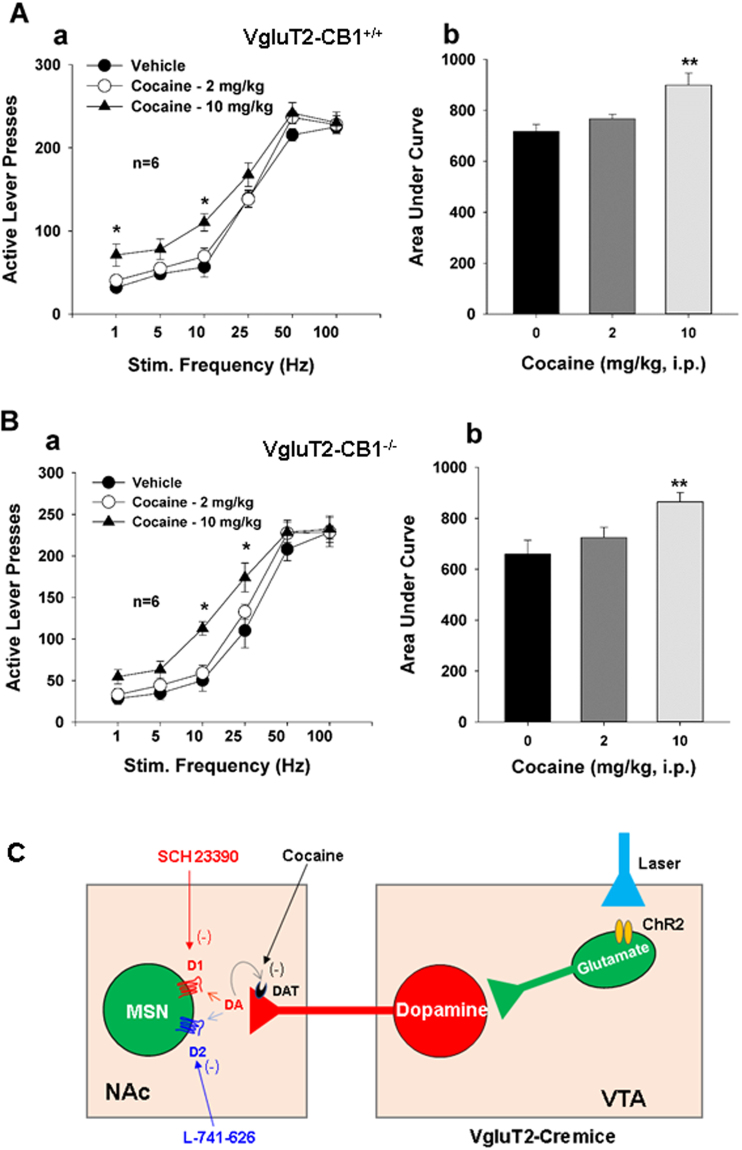
Effects of cocaine on oICSS maintained by activation of VTA glutamatergic neurons. (**A/B**) Systemic administration of cocaine (2, 10 mg/kg, i.p., 15 min prior to testing) dose-dependently shifted the rate-frequency function curve leftward and upward in WT mice (**A-a**) and VgluT2-*CB1* ^−/−^ mice (**B-b**). Calculation of the area under curve (AUC) of the oICSS curve indicates that cocaine-induced leftward and upward shift is statistically significant in both genotypes of mice. (**C**) A schematic diagram indicating how cocaine or D1/D2 receptor antagonists modulate oICSS maintained by optical activation of VTA glutamatergic neurons. *p < 0.05, **p < 0.01, compared to the vehicle treatment group.

To determine whether this glutamate-based oICSS can be used as a new animal model to study brain reward function and the pharmacological action of addictive drugs in the brain, we used the same strategies as used in electrical ICSS in this study: first, we determined whether oICSS displays a typical sigmoid “S”-shape rate-frequency function; and second, we determined whether DA receptor antagonists attenuate, and/or a DA-enhancer (cocaine) enhances oICSS by shifting the rate-frequency curve rightward or leftward, respectively^38^. Figure 5 shows mean rate-frequency function curves, illustrating typical sigmoid “S”-shape curves under vehicle treatment conditions in both wildtype littermates (Fig. 5-A,C) and VgluT2-CB1^−/−^ mice (Fig. 5-B,D). There was no significant difference in oICSS rate-frequency function between the two strains of mice, suggesting that selective deletion of CB1Rs in VgluT2-positive glutamatergic neurons does not alter the basal level of brain reward function. Strikingly, this glutamate-based oICSS was dose-dependently blocked by SCH 23390 (a selective D1 receptor antagonist) or L-741,626 (a selective D2 receptor antagonist), suggesting that the oICSS was mediated indirectly by activation of the mesolimbic DA system (also see Fig. 6C). Two-way ANOVAs for repeated measures over drug dose and stimulation frequency revealed statistically significant drug treatment main effects (Fig. 5A: *F*_1,3_ = 61.04, *p* < 0.01; Fig. 5B: *F*_1,3_ = 280.15, *p* < 0.001; Fig. 5C: *F*_2,6_ = 13.23, *p* < 0.01; Fig. 5D: *F*_2,6_ = 27.39, *p* < 0.001). To further confirm this DA-dependency, we used the same optogenetic approach to directly stimulate VTA DA neurons in DAT-Cre mice. We found that photoactivation of VTA DA neurons induced more robust ICSS behavior than photoactivation of VTA glutamatergic neurons (Fig. 5E: *F*_1,15_ = 22.08, *p* < 0.001), suggesting that VTA glutamate neuron activation-induced oICSS behavior is mediated indirectly by activation of VTA dopaminergic neurons (Figs 5F and 6C).

We then examined whether cocaine produces enhanced brain-stimulation reward effects in oICSS similarly as seen in electrical ICSS^38^. Figure 6 shows that systemic administration of cocaine (2, 10 mg/kg, i.p., 15 min prior to testing) dose-dependently shifted the rate-frequency response curve leftward and upward in both WT and VgluT2-*CB1* ^−/−^ mice. This is consistent with our previous finding with electrical ICSS^38,39^. Two-way ANOVA for repeated measures over cocaine dose and stimulation frequency revealed a significant cocaine treatment main effect in both WT mice (Fig. 6-Aa: *F*_2,10_ = 9.27, *p* < 0.01) and VgluT2-CB1^−/−^ mice (Fig. 6-Ba: *F*_2,10_ = 11.15, *p* < 0.01). One-way ANOVA for repeated measures over cocaine dose revealed a significant increase in the area under curve (AUC) after cocaine administration (Fig. 6-Ab, *F*_2,10_ = 9.26, *p* < 0.01; Fig. 6-Bb, *F*_2,10_ = 11.17, *p* < 0.01), compared to the vehicle treatment group. These data suggest that, in the presence of cocaine, less stimulation strength (Hz) is required to maintain the optical brain-stimulation behavior. Figure 6C shows the proposed mechanisms through which DA receptor antagonists or cocaine modulates oICSS maintained by optical activation of VTA glutamatergic neurons.

### Δ^9^-THC inhibits glutamate-based optical ICSS behavior

**Figure 7:**
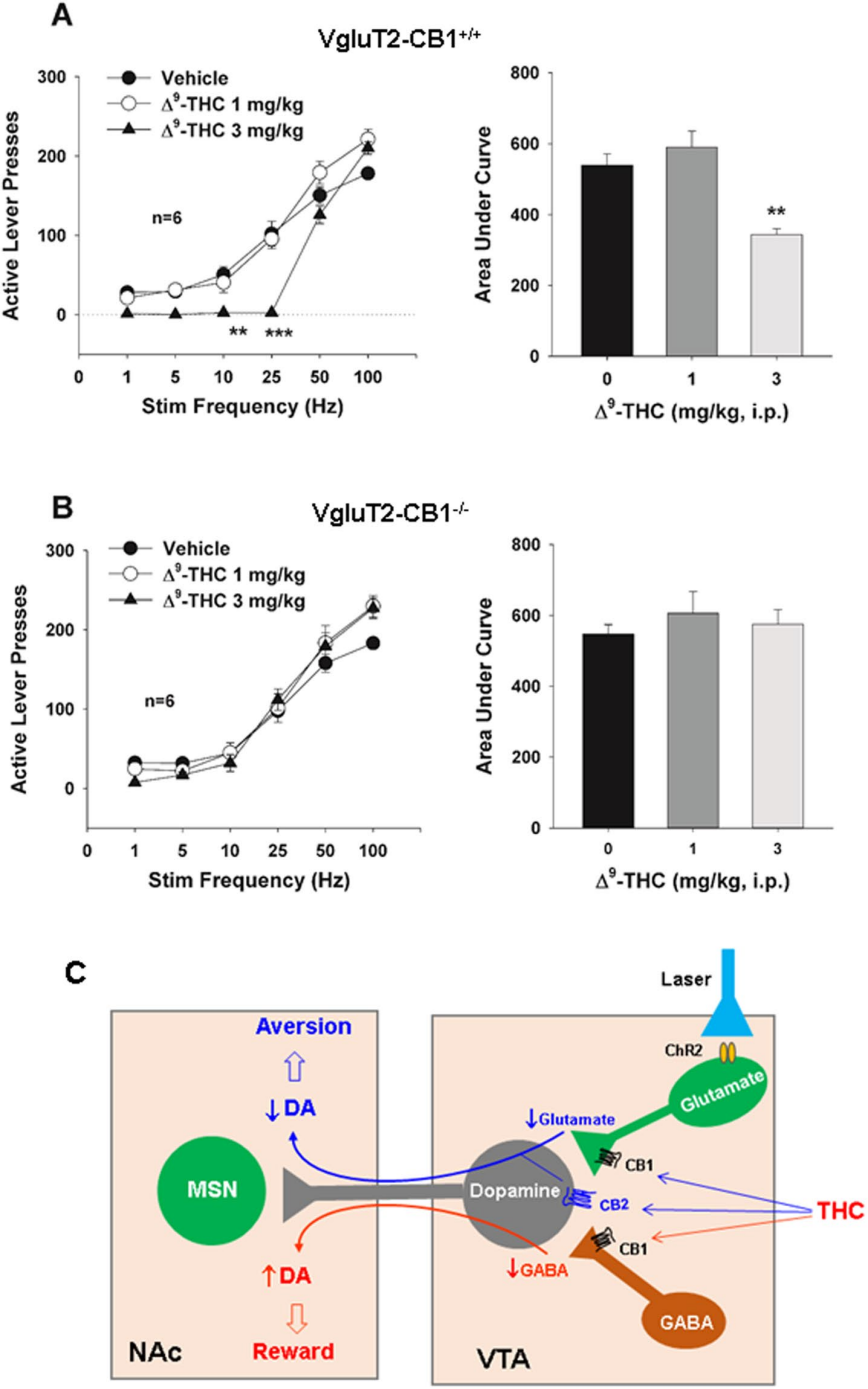
Effects of Δ^9^-THC on oICSS maintained by activation of VTA glutamatergic neurons. (**A/B**) Systemic administration of Δ^9^-THC (1, 3 mg/kg, i.p., 15 min prior to testing) dose-dependently shifted the rate-frequency function curve to the right in WT mice (**A-a**), but not in VgluT2-CB1^−/−^ mice (**B-a**). Calculation of the area under curve (AUC) of the oICSS curve indicates that the Δ^9^-THC-induced rightward shift is statistically significant in WT (**A-b**), not in VgluT2-CB1^−/−^ mice (**B-b**). (**C**) A schematic diagram showing the proposed mechanisms through which cannabis or Δ^9^-THC produces rewarding or aversive effects (see more explanations in the discussion section). **p < 0.01, ***p < 0.001, compared to the vehicle control group in WT mice.

We then examined the effects of Δ^9^-THC on VTA glutamate neuron activation-induced oICSS. Figure 7 shows that Δ^9^-THC dose-dependently shifted the rate-frequency function curve downward and rightward only in WT mice (Fig. 7A-a), but not in VgluT2-CB1^‒/‒^ mice (Fig. 7B-a). Two-way ANOVA for repeated measures over Δ^9^-THC dose and stimulation frequency revealed a statistically significant Δ^9^-THC treatment main effect (Fig. 7A-a: *F*_2,10_ = 15.05, *p* < 0.001) in WT mice, but not in VgluT2-CB1^−/−^ mice (Fig. 7B-a, *F*_2,10_ = 1.001, *p* > 0.05). These findings suggest that, in the presence of Δ^9^-THC, higher stimulation strength (Hz) is required to maintain the optical ICSS behavior. We note that Δ^9^-THC appears more effective in inhibiting optical ICSS maintained by low-frequency (1, 5, 10, 25 Hz) than by high-frequency (50, 100 Hz) optical stimulation (Fig. 7A-a), suggesting that the effects of Δ^9^-THC are reversed by increasing VTA glutamate release or stimulation strength. Figure 7C shows a schematic diagram illustrating that activation of CB1Rs in VTA glutamatergic neurons may mediate Δ^9^-THC-induced aversion since selective deletion of CB1Rs in glutamatergic neurons in VgluT2-CB1^−/−^ mice attenuates Δ^9^-THC-induced reduction in oICSS in this study.

### Δ^9^-THC inhibits basal and glutamate-enhanced locomotion

**Figure 8:**
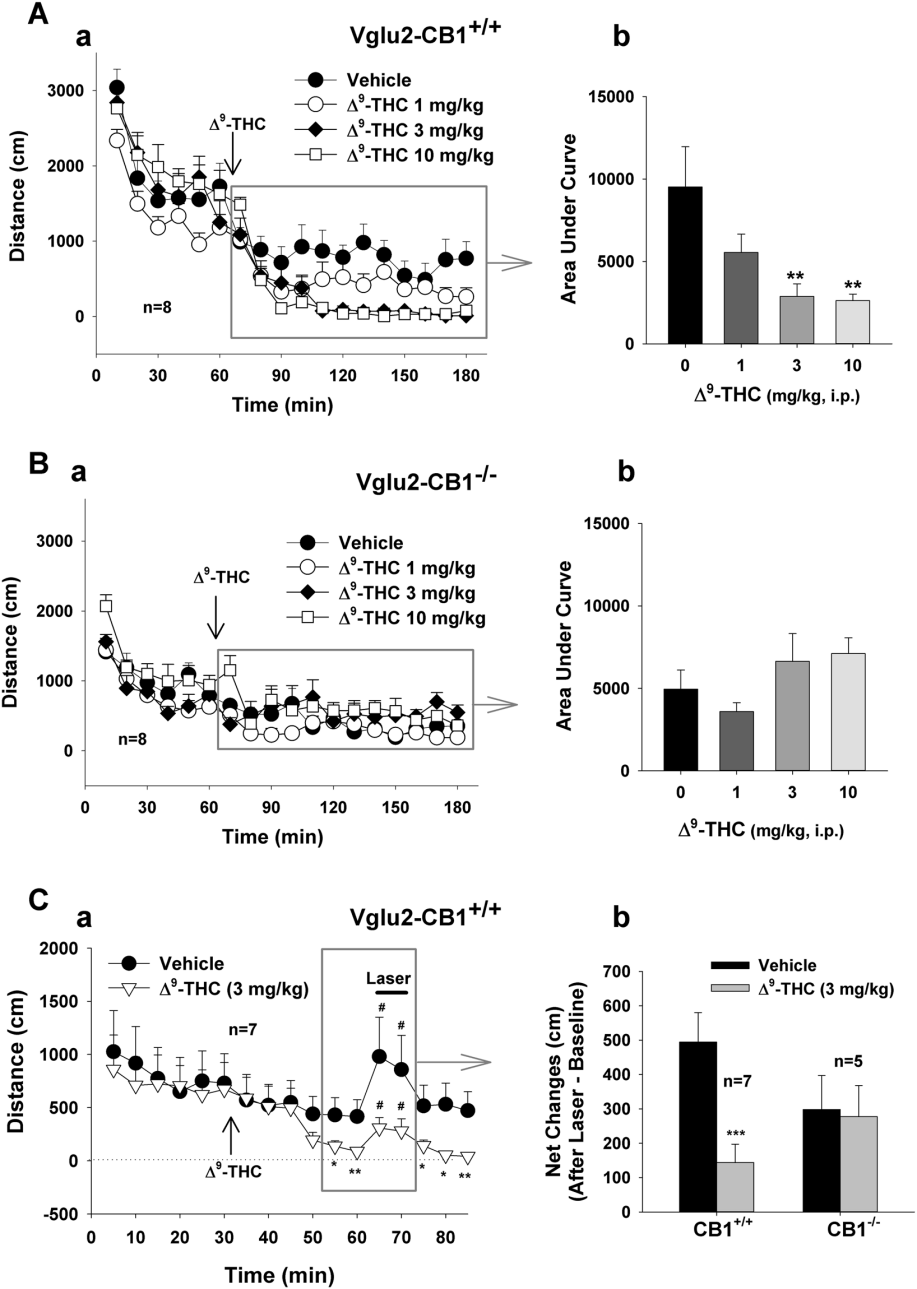
Effects of Δ^9^-THC on basal and VTA glutamate neuron activation-enhanced locomotion. (**A**,**B**) Systemic administration of Δ^9^-THC (1, 3, 10 mg/kg, i.p., 15 min prior to testing) dose-dependently inhibited basal locomotion in WT mice (**A**), but not in VgluT2-*CB1* ^−/−^ mice (**B**). Calculation of the area under curve (AUC) of locomotor activity indicates a significant reduction in locomotion after 3 or 10 mg/kg Δ^9^-THC administration in WT (**A-b**), not in VgluT2-CB1^−/−^ mice (**B-b**). (**C**) Effects of Δ^9^-THC pretreatment on locomotor response to photoactivation of VTA glutamatergic neurons, indicating that 3 mg/kg Δ^9^-THC also significantly inhibited photo-stimulation-enhanced locomotor response in WT mice (**C-a**), but not in VgluT2-*CB1* ^−/−^ mice (**C-b**). *p < 0.05, **p < 0.01, ***p < 0.001, compared to the vehicle control group (**A-b, C-b**) or the baseline before Δ^9^-THC administration (**C-a**). ^#^ *p* < 0.05, compared to the baseline before laser stimulation (**C-a**).

Finally, we examined whether the inhibitory effects of Δ^9^-THC on oICSS generalize to other actions of Δ^9^-THC. Figure 8-A,B shows that systemic administration of Δ^9^-THC (1, 3, 10 mg/kg, i.p.) significantly decreased basal levels of locomotion in a dose-dependent manner in WT littermates (Fig. 8A), but not in VgluT2-CB1^‒/‒^ mice (Fig. 8B). One-way ANOVA for repeated measures over drug dose revealed a statistically significant treatment main effect in WT mice (Fig. 8A-b: *F*_3,18_ = 8.51, *p* < 0.001), but not in VgluT2-CB1^‒/‒^ mice (Fig. 8B-b: *F*_3,18_ = 2.44, *p* > 0.05). Unexpectedly, VgluT2-CB1^‒/‒^ mice displayed a significantly lower basal level of locomotion (before Δ^9^-THC administration) than WT mice (Fig. 8A *versus* Fig. 8B), suggesting that CB1R expression in VgluT2-positive glutamate neurons contributes to maintaining basal locomotor activity.

Figure 8-Ca shows that Δ9-THC, at 3 mg/kg, not only decreased basal locomotion, but also significantly attenuated the locomotor response to photostimulation of VTA glutamate neurons.

Figure 8-Cb shows the mean net increase in distance traveled (cm per 5 min) after VTA glutamate neuron activation, indicating a significant reduction in evoked locomotor response after Δ9-THC administration in WT littermates (*F*1,6 = 16.88, *p* < 0.001), but not in VgluT2-CB1^‒/‒^ mice (*F*_1,4_ = 0.38, *p* > 0.05).

## Discussion

The major findings in the present study include: 1) CB1R mRNA was detected in both VTA glutamatergic neurons and GABAergic neurons with the density higher in glutamatergic neurons than in GABAergic neurons; 2) systemic administration of Δ^9^-THC produced significant conditioned place aversion in wildtype littermates, but not in VgluT2-*CB1* ^−/−^ mice; 3) optical activation of VTA glutamatergic neurons is rewarding, as assessed by optical ICSS, which was dose-dependently enhanced by cocaine, but attenuated by DA receptor antagonists, suggesting DA-dependent reward substrate; 4) Δ^9^-THC dose-dependently inhibited VTA glutamate neuron activation-induced optical ICSS in wildtype littermates, but not in VgluT2-*CB1* ^−/−^ mice, suggesting an effect mediated by activation of CB1Rs in VgluT2-expressing glutamate neurons; and finally, 5) Δ^9^-THC also inhibited basal or VTA glutamate neuron activation-enhanced locomotion. Taken together, the present findings indicate for the first time that endocannabinoid mechanisms associated with VgluT2-expressing glutamatergic neurons mediate hedonic and locomotor effects of Δ^9^-THC in mice.

With regard to the present cannabinoid findings, it is well known that cannabis can be rewarding or dysphoric in both humans and experimental animals^24,25^. Electrical ICSS is a commonly used behavioral procedure to study brain reward functions. In this model, animals press a lever to deliver brief electrical pulses to a discrete brain region via an implanted electrode. Most addictive drugs lower the stimulation threshold for electrical ICSS, indicating enhanced rewarding efficacy of the ICSS and implying a summation between the rewarding effects of the electrical ICSS and the pharmacological rewarding effects of the addictive drugs. However, the effects of cannabinoids on brain reward has been a matter of debate. In some studies, Δ^9^-THC produced a significant reduction in the electrical ICSS threshold in rats^39,40^, suggesting enhanced brain-stimulation reward (BSR). However, in other studies, Δ^9^-THC or other cannabinoid agonists either had no effect on electrical BSR^41^ or produced a reduction in electrical ICSS (i.e., aversion) in rats^42–44^. Conflicting findings have also been reported in studies using the CPP model [see reviews by^24,25^]. In the intravenous drug self-administration models, Δ^9^-THC does not support robust self-administration in rodents^24,25^ or rhesus monkeys^26,27^, although Δ^9^-THC was reported to be able to maintain intravenous self-administration in squirrel monkeys^22,23^. The mechanisms underlying such conflicting findings are unclear. Since VTA DA neurons receive both excitatory glutamatergic and inhibitory GABAergic inputs^16,30,45^, we hypothesized that the hedonic effects of cannabis may depend on the final net effect of two opposing actions or different CB1R distributions in both VTA GABAergic and glutamatergic neurons and their afferent terminals (Fig. 7C). If more CB1Rs are expressed in VTA GABAergic neurons or GABAergic afferents), cannabis will be rewarding since GABAergic disinhibition of VTA DA neurons is dominant. In contrast, if more CB1Rs are expressed in VTA glutamatergic neurons or glutamatergic afferents, cannabis will be aversive since CB1R activation-induced reduction of glutamatergic inputs to VTA DA neurons is dominant. Congruently, if CB1R levels are equivalent on both neuronal types, cannabis will have no effect on brain reward function (Fig. 7C). This hypothesis may well explain why Δ^9^-THC or cannabinoids are rewarding in some species, for example, in squirrel monkeys in which more CB1Rs might be expressed in VTA GABAergic neurons or GABAergic afferent terminals, but are ineffective or aversive in other species such as rats and mice in which more CB1Rs might be expressed in VTA glutamatergic neurons or glutamatergic afferent terminals.

Early autoradiography, IHC and ISH assays indicated that CB1Rs are mainly expressed in cerebral cortex, hippocampus, globus pallidus, substantia nigra pars reticulata, and cerebellum, while a low density of CB1Rs is found in the VTA^11,46–48^. Although CB1R-immunostaining was detected in GABAergic interneurons in hippocampus, hypothalamus and cerebellum^11,49,50^, little anatomic evidence indicates whether CB1Rs are expressed in VTA GABAergic interneurons or glutamatergic neurons. In addition, the manner by which cannabis produces anhedonic or aversive effects has remained elusive. In the present study, we used double-labeling highly sensitive RNAscope ISH assays indicating that CB1R mRNA is expressed in both glutamatergic and GABAergic neurons within the VTA, but with higher densities in VTA glutamatergic neurons than in VTA GABAergic neurons, which provides the first direct evidence suggesting that CB1Rs in glutamatergic neurons may play a dominant role in controlling VTA DA neuron activity. This finding may also explain why Δ^9^-THC and other CB1 agonists are generally not rewarding, but aversive in rodents as reported previously^24,25,32^ and in the present study.

Another important finding is that the aversive effects of Δ^9^-THC are mediated by activation of CB1Rs in VgluT2-expressing glutamatergic neurons. This conclusion is supported by several lines of evidence. First, Δ^9^-THC produced conditioned place aversion in wildtype littermates, but not in VgluT2-*CB1* ^−/−^ mice; second, Δ^9^-THC significantly inhibits brain-stimulation reward produced by optical activation of VTA glutamatergic neurons only in wildtype littermates, but not in VgluT2-*CB1* ^−/−^ mice; third, Δ^9^-THC significantly inhibits basal levels of locomotion by activation of CB1Rs in VgluT2-expressing glutamatergic neurons; and lastly, higher densities of CB1R mRNA were found in VTA glutamatergic neurons than in VTA GABAergic neurons. Together, these findings are consistent with a recent report that selective deletion of CB1Rs in cortical principal (possibly glutamatergic) neurons, but not in GABAergic neurons, significantly attenuated Δ^9^-THC’s classical “tetrad effects” – analgesia, hypothermia, catalepsy, and hypolocomotion^51^.

We note that selective deletion of CB1Rs in VgluT2-expressing glutamatergic neurons significantly attenuated Δ^9^-THC-induced place aversion, but it did not reveal conditioned place preference in VgluT2-*CB1* ^‒/‒^ mice. In other words, in the absence of CB1Rs in VTA glutamatergic neurons or glutamatergic afferents terminals, Δ^9^-THC should produce CPP or enhanced oICSS in VgluT2-*CB1* ^‒/‒^ mice. But this is not the case in the present study. There are two possible explanations. First, VTA DA neurons may also receive VgluT1-expressing glutamatergic inputs from other brain regions, where high densities of CB1Rs are also expressed^11,46,50^; second, we have recently reported that cannabinoid CB2Rs are also expressed in VTA DA neurons and functionally inhibit VTA DA neuron activity and DA-related behavior in both rats and mice^52,53^. Thus, activation of CB1Rs in VgluT1-expressing glutamatergic terminals and/or CB2Rs in VTA DA neurons may antagonize the rewarding effects produced by CB1R activation in GABAergic neurons (Fig. 7C). We also note that Δ^9^-THC, at 3 mg/kg, did not inhibit high-frequency optical stimulation-induced ICSS, suggesting that higher Δ^9^-THC doses may be required. However, the locomotor inhibition produced by higher doses of Δ^9^-THC prevented testing higher doses of Δ^9^-THC in this behavioral study. Although Δ^9^-THC also dose-dependently inhibited levels of locomotion during periods of no optical stimulation, suggesting potential sedative effects, the reduction in optical ICSS is unlikely due to Δ^9^-THC-induced sedation or locomotor impairment since 3 mg/kg Δ^9^-THC failed to inhibit oICSS in both WT and VgluT2-*CB1* ^‒/‒^ mice. The finding that Δ^9^-THC also inhibits basal locomotion in wildtype littermates, but not in VgluT2-*CB1* ^−/−^ mice, provides additional evidence that CB1Rs on VgluT2-expressing glutamatergic neurons play an important role in mediating cannabis-mediated rewarding and psychomotor-stimulating actions in mice.

Surprisingly, VgluT2-*CB1* ^−/−^ mice displayed a significant reduction in basal levels of locomotion, which seemingly conflicts with the finding that activation of CB1Rs by Δ^9^-THC inhibits locomotion. The present finding observed in conditional VgluT2-*CB1* ^‒/‒^ mice is consistent with that in constitutive CB1-knockout mice (i.e., global *CB1* ^‒/‒^) that display a significant reduction in basal locomotion and in locomotor/DA responses to cocaine compared to WT mice^54^. One possible interpretation is that endocannabinoids affect basal levels of locomotion not only through CB1Rs in the mesolimbic DA system, but also through CB1Rs in acetylcholine or other locomotion-related neurotransmitter systems. The reduction in basal locomotion observed in VgluT2-*CB1* ^‒/‒^ mice could be a final net effect of multiple actions in different neurotransmitter systems.

A third important discovery is that oICSS may be used as a new animal model to study cell type-specific brain reward mechanisms. In this study, we used the same criteria as used in electrical ICSS^38,44^, we successfully established a stable rate-frequency function curve of oICSS produced by optical activation of VTA glutamatergic neurons. We found that systemic administration of cocaine significantly enhanced oICSS as assessed by a leftward shift of the rate-frequency curve. This is consistent with findings observed in electrical ICSS^38^. In contrast, DA receptor antagonists significantly inhibited oICSS and shifted the rate-frequency curve to the right, suggesting that the oICSS produced by activation of VTA glutamate neurons is mediated indirectly by activation of VTA DA neurons. This is further supported by the finding that VTA axon terminals expressing VgluT2 form synapses on VTA DA neurons, and photoactivation of VTA VgluT2 neurons supports oICSS and produces conditioned place preference by increasing glutamate release onto VTA DA neurons^34^. Since oICSS is mediated by selective activation of a specific phenotype of neurons in the brain, it is superior to electrical ICSS in studying cell type-specific neural circuitries underlying brain reward or aversion as well as pharmacological action of a test compound.

Although CB1 mRNA was detected in both VTA glutamatergic neurons and GABAergic neurons, it is unlikely that the present findings reflect CB1R mechanisms only within the VTA, since higher densities of CB1Rs are expressed in other brain regions outside the VTA^9,11,46^. Given that VTA DA neurons also receive glutamatergic afferents from the medial prefrontal cortex, the pedunculopontine region, and the subthalamic nucleus^13,55^; and VTA DA neurons also receive GABAergic afferents from the ventral striatum, ventral pallidum, and rostromedial tegmental nucleus (RMTg)^15^, the aversive effects of Δ^9^-THC observed in the present study are most likely the final outcome of opposing actions in VTA GABAergic *versus* glutamatergic neurons and/or GABAergic *versus* glutamatergic afferents from other brain regions.

In the present study, we did not examine the role of CB1Rs in VTA GABAergic neurons or GABAergic afferents in cannabis reward since Δ^9^-THC is not rewarding in mice as assessed by the mouse CPP model. The presently-used oICSS techniques cannot be used to address this question since optical activation of VTA GABAergic neurons in Vgat-Cre mice cannot induce motivated ICSS behavior as shown in our pilot study. This is not surprising since optical activation of VTA GABAergic neurons increases GABA release that, in turn, inhibits VTA DA neurons (Fig. 7C). Therefore, additional behavioral models, involving conditional CB1^-/-^ mice in brain GABAergic neurons, are required to further address the above GABAergic hypothesis of cannabis reward.

In conclusion, the present findings demonstrate for the first time that CB1Rs in cell type-specific (VgluT2-expressing) glutamatergic neurons play an important role in the hedonic effects of Δ^9^-THC. Given that optical ICSS is mediated by direct activation of VTA dopaminergic or glutamatergic neurons and displays a similar sigmoid “S”-shape rate-frequency function as seen in classical electrical ICSS, oICSS may be used as a new animal model to study cell type-specific neural circuitries underlying brain reward and aversion, and the pharmacological actions of addictive or anti-addictive drugs in the brain.

## Methods

### Animals

Adult VgluT2-*CB1* ^−/−^ mice (CB1^flox/flox;VgluT2-*Cre*+/−)^ and their wild-type littermates (VgluT2-*Cre* ^+/−^), aged 4–16 weeks, were used in the all behavioral experiments, and maintained on a 12 h light-dark cycle with food and water available *ad libitum*. All experimental procedures were conducted in accordance with the *Guide for the care and use of laboratory animals* of the U.S. National Research Council and were approved by the animal care committee of the National Institute on Drug Abuse of the National Institutes of Health. More details about the generation of conditional CB1 knockout (cKO) mice were provided in the Supplement.

### Immunohistochemistry Assays

We used fluorescent immunochemistry to examine CB1R expression in the VTA. Methods in d﻿etail are described in the Supplementary Information.

### RNAscope ***in situ*** hybridization assays

All the RNA probes that target CB1, VgluT2 or GAD1 were designed and provided by Advanced Cell Diagnostics (Hayward, CA). Complete experimental methods are described in the Supplementary Information.

### Conditioned place preference or aversion

Classical conditioned place preference experimental procedures were as described previously (Xi *et al*., 2011). A three-chamber place preference apparatus (Med Associates) was used in this study. Three groups of wild-type mice (*n* = 12 each group) and two groups of VgluT2-*CB1* ^−/−^ mice were used to study Δ^9^-THC-induced (1, 3, 5 mg per kg) conditioned place preference or aversion. More details are provided in the Supplementary Information.

### Optical intracranial self-stimulation

For oICSS experiments, mice were injected with AAV-EF1α-DIO-ChR2-EGFP) or the control virus (AAV2-EF1α-DIO-EGFP) and implanted with bilateral custom-made optical fiber targeted to the VTA. Behavioral training and testing occurred in mouse operant chambers (Med Associates) interfaced with optogenetic stimulation equipment. Complete experimental methods are described in the Supplementary Information.

### Data Analysis

All data are presented as mean ± SEM. One-way ANOVA or two-way ANOVA for repeated measures was used to analyze the difference between WT and VgluT2- *CB1* ^−/−^ mice in terms of active lever presses, or travel distance. Paired comparisons between WT and VgluT2-*CB1* ^−/−^ groups were carried out using the Student–Newman–Keuls method.

## Electronic supplementary material

Suppl Infor
