# Supplementary material for: CB1 Receptor Activation on VgluT2-Expressing Glutamatergic Neurons Underlies Δ9-Tetrahydrocannabinol (Δ9-THC)-Induced Aversive Effects in Mice
Source: Sci Rep. 2017 Sep 26;7:12315. doi: 10.1038/s41598-017-12399-z (PMC5614984; doi:10.1038/s41598-017-12399-z)
Supplement: Supplementary file 1 — Suppl Infor [file 41598_2017_12399_MOESM1_ESM.pdf]

Scientific Reports

CB1 Receptor Activation on VgluT2-Expressing Glutamatergic Neurons Underlies Δ^9-Tetrahydrocannabinol (Δ^9-THC)-Induced Aversive Effects in Mice

Xiao Han[1,2,*], Yi He[1,*], Guo-Hua Bi[1], Hai-Ying Zhang[1], Rui Song[2], Qing-Rong Liu[3], Josephine M. Egan[3], Eliot L. Gardner[1], Jing Li[2,#], Zheng-Xiong Xi[1,#]

Online Methods

Animals.

Due to the unavailability of CB1$^{flox/flox}$ mice in North America, we used similar strategies as reported previously (*1*) to generate this mouse line for the present study. Adult VgluT2-*CB1*$^{-/-}$ mice (CB1$^{flox/flox;VgluT2\text{-}Cre+/-}$) and their wild-type littermates (VgluT2-*Cre*$^{+/-}$), aged 4-16 weeks, were used in the all behavioral experiments, and maintained on a 12 h light-dark cycle with food and water available *ad libitum*. All experimental procedures were conducted in accordance with the *Guide for the care and use of laboratory animals* of the U.S. National Research Council. Conditional CB1 knockout (cKO) mice were obtained by using the Cre/loxP system. VgluT2-(*Slc17a6*$^{tm2(cre)Lowl}$, stock #016963)-IRES-Cre (i.e., VgluT2-Cre) knock-in mice were obtained from the Jackson Laboratory (# 016963) and then bred at the National Institute on Drug Abuse (NIDA), Intramural Research Program (IRP). VgluT2-*CB1*$^{-/-}$ mice were generated by crossing VgluT2-Cre mice with CB1-floxed (*CB1*$^{flox/flox}$) mice, using a three-step breeding protocol. VgluT2-*CB1*$^{-/-}$ mice lack the CB1 receptor mainly in subcortical glutamatergic neurons expressing the vesicular glutamate transporter 2 (VgluT2). The mutant lines were bred for >10 generations on the background of C57BL/6 mice from Charles River Laboratories (Frederick, MD, USA). Genotyping for the Cre transgene was performed by PCR using the following primers: forward (5′ - GAT CGC TGC CAG GAT ATA CG); reverse (5′ - CAT CGC CAT CTT CCA GCA G), while genotyping for the *CB1*$^{flox/flox}$ locus was performed by PCR using the following primers: forward (5′ - CCT AAG AAC TGC ATG GCA TGA AG), reporter-1 (5′ - CTA GCA TCT GTT GGA GTG TAC); reverse (5′ - GGA ACT TCG CTA GAC TAG TAC GC).

Animal surgeries

Male mice (~4 weeks of age) were anesthetized with sodium pentobarbital (70 mg/kg, i.p.) and placed in a stereotaxic frame (David Kopf Instruments, Tujunga, CA, USA). For intra-VTA microinjection of virus, a custom-made 30-guage stainless injector was used to infuse Cre-inducible recombinant adeno-associated virus (AAV) that encodes channelrhodopsin-2 (ChR2) and enhanced green fluorescent protein (EGFP) (i.e., AAV- EF1α-DIO-ChR2-EGFP) or the control virus (AAV2-EF1α-DIO-EGFP) (300 nl, ~2×10^{12} genomes/ml, University of North Carolina Gene Therapy Center) unilaterally into the VTA (AP -3.2; ML 0.1; DV -4.2 mm relative to Bregma) using a micropump (WPI 2000 UltraMicroPump, Sarasota, FL, USA) with a speed of 50 nl/min. For optical brain stimulation, a custom-built optrode (200-μm multimode optical fiber, Thorlabs, Newton, NJ, USA) tethered to an intracranial ceramic ferrule (MM-FER2007C-2300, Precision Fiber Products, Inc., Milpitas, CA, USA) was implanted into the VTA (*AP* −0.32; *ML* 0.1, *DV* −3.7 mm relative to Bregma) at the AAV injection site. Dental cement was used to fix the optrode assembly to the skull. Following AAV vector injection and optrode implantation, mice were allowed to recover for at least 2 weeks before optical self-stimulation experiments began.

Conditioned place preference or aversion

Three groups of wild-type mice (*n* =12 each group) and two groups of VgluT2-$CB1^{-/-}$ mice were used to study Δ^9-THC -induced (1, 3, 5 mg per kg) conditioned place preference or aversion. A three-chamber place preference apparatus (Med Associates) was used in this study. The apparatus consisted of two large compartments (16.8 × 12.7 × 12.7 cm) and one small compartment (7.2 × 12.7 × 12.7 cm), which separated the large compartments. The two large

compartments had different visual and tactile cues. One compartment was black with a stainless steel rod floor. The other compartment was white with a stainless steel mesh floor. The small compartment was gray with a smooth polyvinyl chloride floor. The apparatus had a clear Plexiglas top with a light on it. During the preconditioning phase (days 1–2), mice were placed in the small compartment and were allowed to freely explore the three compartments for 15 min daily. The time spent in each compartment was recorded. We used an unbiased conditioned place preference procedure. Mice spending over 500 s in either compartment were excluded. The next 7 d (days 3–9) constituted the conditioning phase, with one session per day. Each mouse received an intraperitoneal injection of the same dose of Δ^9-THC on days 3, 5, 7 and 9, and was then confined in one large compartment for 45 min. On days 4, 6, and 8, each mouse received an intraperitoneal injection of vehicle (5% Cremophore) and was then confined in the other large compartment for 45 min. Test drug was paired with either white or black compartment in a counterbalanced manner. On the test days (24 h and 48h after the last Δ^9-THC injection), mice were allowed to freely explore the three compartments for 15 min without injections, and the time spent in each compartment was recorded. All behavioral testing was performed during the light phase of the light/dark cycle. The mean CPP scores (i.e., time spent in Δ^9-THC -paired compartment – time spent in vehicle-paired compartment) over the two test days were used to compare the rewarding effects of Δ^9-THC between two strains of mice.

Optical intracranial self-stimulation (oICSS) apparatus

Optical stimulation experiments were conducted in standard operant conditioning chambers (Med Associates, Fairfax, VT, USA). Each chamber was equipped with two wall-mounted levers, two cue lamps, a house lamp, an audio stimulus generator, and four pairs of infrared

detectors. Mice were gently connected to a cable that was in turn connected to a 473 nm laser tuned for ChR2 stimulation via an optical swivel. Computer software controlled a pulse generator that controlled the lasers.

oICSS Procedure

The general procedures for optical ICSS were modified based on those reported previously in optical ICSS (*2*, *3*) and electrical ICSS experiments(*4*). After 2 weeks of recovery from surgery, mice were placed into operant chambers containing two operant levers – an active lever and an inactive lever, respectively (ENV-307W-CT, Med associates Inc., Fairfax, VT, USA). The optrode implanted into the mouse brain (VTA) was connected to a 473 nm laser (OEM Laser Systems, Inc., Draper, UT, USA) via an optical swivel (Doric Lenses Inc, Quebec, Canada). Animals were initially trained on a fixed-ratio 1 (FR1) reinforcement schedule; each active lever response led to delivery of a 1-s pulse train of light stimulation (473 nm, 20 mW, 5 ms duration, 25 Hz) accompanied by a 1-s illumination of cue light above the lever. While inactive lever presses were counted, they had no programmed consequence. Each daily training session lasted 60 min. An additional group of mice received a lever switch test in which the assignment of active and inactive levers with respect to the right and left levers was reversed to confirm that the lever responding was photostimulation reward-contingent.

Rate-frequency oICSS procedure

Following establishment of lever-pressing for oICRS, animals were presented with a series of 6 different stimulation frequencies (100, 50, 25, 10, 5, 1 Hz) in descending order to obtain rate-frequency response curves. Animals were allowed to respond for 10 min per stimulation

frequency. The animals were then divided into 3 groups (4-6 mice per group) to observe the effects of DA receptor antagonists (SCH23390, 0.2 mg/kg, i.p.; L-741,626, 3 or 10 mg/kg, i.p., 10 min prior to testing), cocaine (0, 2, 10 mg/kg, i.p.), or Δ^9-THC (vehicle, 1, 3 mg/kg, i.p.), respectively, on optical BSR maintained by photostimulation of VTA glutamate neurons in VgluT2-Cre mice. Each animal received 3-5 drug injections during the oICSS experiments. After each test, animals received an additional 5-7 days of oICSS re-stabilization until a new baseline of lever responding was established. The order of testing for the various doses of the drugs was counterbalanced. The effects of DA receptor antagonists, cocaine, or Δ^9-THC on oICSS were evaluated by comparing drug-induced changes in active lever presses in VgluT2-Cre mice. All the oICSS experiments were performed in VgluT2-Cre mice, while an additional group of DAT-Cre (DAT-CB1$^{+/+}$) mice were used to compare oICSS maintained by photoactivation of VTA dopaminergic neurons in DAT-Cre mice or by photoactivation of VTA glutamatergic neurons in VgluT2-Cre mice.

Locomotor activity

This experiment was designed to compare the effects of Δ^9-THC on basal and VTA glutamate-enhanced locomotion (i.e., locomotor response to photostimulation of VTA glutamate neurons) between WT littermates and VgluT2-*CB1*$^{-/-}$ mice. After completion of the above optical ICSS experiment, mice were placed in open-field locomotor chambers (Accuscan, Columbus, OH, USA) and habituated for 1 h. After 3 days of habituation, mice were then divided into 2 groups (5-8 mice per group). One group was used to determine the effects of Δ^9-THC on basal levels of locomotion, in which each animal randomly received vehicle or one dose of Δ^9-THC (1, 3, 10 mg/kg, i.p.) with 2-4 days of intervals. Another group of mice was used to observe the effects

of Δ^9-THC on VTA glutamate-enhanced locomotion. Following each injection, locomotor activity was recorded for 2 h in 5 or 10 min bins, and the traveled distance was used to evaluate the effects of Δ^9-THC on basal locomotion or photostimulation-evoked locomotion.

Immunohistochemistry assays

After completion of the above behavioral experiment, animals were anesthetized with sodium pentobarbital (100 mg/kg, i.p.) and intracardially perfused with ice-cold 0.9% saline followed by 4% paraformaldehyde. Brains were coronally sectioned at 30 μm. The brain sections were processed for immunohistochemistry assays to observe VTA tyrosine hydroxylase (TH) or AAV-EGFP expression as reported previously (Song et al., 2012). Briefly, free-floating coronal sections from VgluT2-EF1α-ChR2-EGFP mice (*n* =4) were incubated for 1 h in PB supplemented with 4% bovine serum albumin and 0.3% Triton X-100. Sections were then incubated with cocktails of primary antibodies [rabbit polyclonal anti-CB1 (Abcam, # ab23703; 1:500) or chicken anti-GFP (Aves Lab Inc., #GFP1020; 1:500) + mouse polyclonal anti-TH antibody (Millipore, #MAB318; 1:500)] overnight at 4 °C. After three rinses (10 min each) in phosphate buffer, sections were incubated in a cocktail of the corresponding fluorescently labeled secondary antibodies (Alexa Fluor 594 goat anti-mouse and Alexa Fluor 488 anti-chicken) (1:500; Life Technologies) for 1 h at room temperature. After rinsing, sections were mounted on gelatin-coated slides with 50% buffered glycerol. Mounted sections were coverslipped with DAPI nuclear counterstain (H-1200, Vector Laboratories). Fluorescent images were collected with an Olympus FV1000 Confocal System (Olympus, Tokyo, Japan) using manufacturer-provided software.

RNAscope *in situ* hybridization (ISH)

RNAscope ISH was used to detect cell type-specific CB1 mRNA expression in VgluT2-CB1$^{-/-}$ mice (CB_1flox$^{+/+}$, VgluT2$^{+/-}$) and their wild-type littermates (CB1$^{flox/flox}$). Mice were deeply anesthetized and the whole brain was removed and rapidly frozen on dry ice. Fresh-frozen tissue sections (14 µm thick) were mounted on positively charged microscopic glass slides (Fisher Scientific) and stored at -80 °C until RNAscope ISH assays could be performed. Multiple target gene-specific RNAscope probes were used to observe the cellular distributions of CB1 mRNA in VgluT2-expressing glutamate neurons, TH-expressing DA neurons, and Gad1-expressing GABAergic neurons: CB1 RNAscope probe (Cat #: 420721, targeting 530–1,458 bp of the mouse Cnr1 mRNA sequence, NM_007726.3,), VgluT2 RNAscope probe (Cat #: 319171-C3, targeting 1,986–2,998 bp of the Mus musculus VgluT2 mRNA sequence, NM_080853.3), TH-specific RNAscope probe (Cat #: 317621-C2, targeting 483–1,603 bp of the Mus musculus TH mRNA sequence, NM_009377.1), and Gad1-specific RNAscope probe (Cat#: 400951-C3, targeting 62-3113 bp of NM_008077.4). All these probes were designed and provided by Advanced Cell Diagnostics. The RNAscope mRNA-staining steps were performed following the manufacturer's protocols. Stained slides were cover-slipped with fluorescent mounting medium (ProLong Gold Anti-fade Reagent P36930; Life Technologies) and scanned into digital images with an Olympus FluoView FV1000 confocal microscope at 40× or 60× magnification using manufacturer-provided software. The CB1 mRNA-signal densities and the detected object counting in individual VgluT2-positive or Vgat-positive neurons are as reported previously (*5*).

Drugs

Cocaine HCl and Δ^9-tetrahydrocannabinol (Δ^9-THC) were provided by NIDA IRP. The stock Δ^9-THC solution is 50 mg/ml (w/v) solution in 100% ethanol. The vehicle used to dilute Δ^9-THC is

5% Cremophore (C5135, Sigma-Aldrich). SCH 23390 (D1 receptor antagonist) and L-741,626 (D2 receptor antagonist) were obtained from Tocris Biosciences.

Statistical Analysis

All data are presented as means ± SEM. Data analysis was performed with Prism 5 (GraphPad Software Inc.). One-way or two-way ANOVAs for repeated measures over drug dose and stimulation frequency were used to analyze the significance of the effects after each drug treatment. Post-hoc individual group comparisons were made using the Student–Newman–Keuls method.

References

1. G. Marsicano *et al.*, The endogenous cannabinoid system controls extinction of aversive memories. *Nature* **418**, 530-534 (2002).
2. H. L. Wang, J. Qi, S. Zhang, H. Wang, M. Morales, Rewarding Effects of Optical Stimulation of Ventral Tegmental Area Glutamatergic Neurons. *J Neurosci* **35**, 15948-15954 (2015).
3. A. Ilango *et al.*, Similar roles of substantia nigra and ventral tegmental dopamine neurons in reward and aversion. *J Neurosci* **34**, 817-822 (2014).
4. Z. X. Xi *et al.*, Cannabinoid CB1 receptor antagonists attenuate cocaine's rewarding effects: experiments with self-administration and brain-stimulation reward in rats. *Neuropsychopharmacology* **33**, 1735-1745 (2008).
5. T. M. Grabinski, A. Kneynsberg, F. P. Manfredsson, N. M. Kanaan, A method for combining RNAscope in situ hybridization with immunohistochemistry in thick free-floating brain sections and primary neuronal cultures. *PLoS One* **10**, e0120120 (2015).

Figure S1:

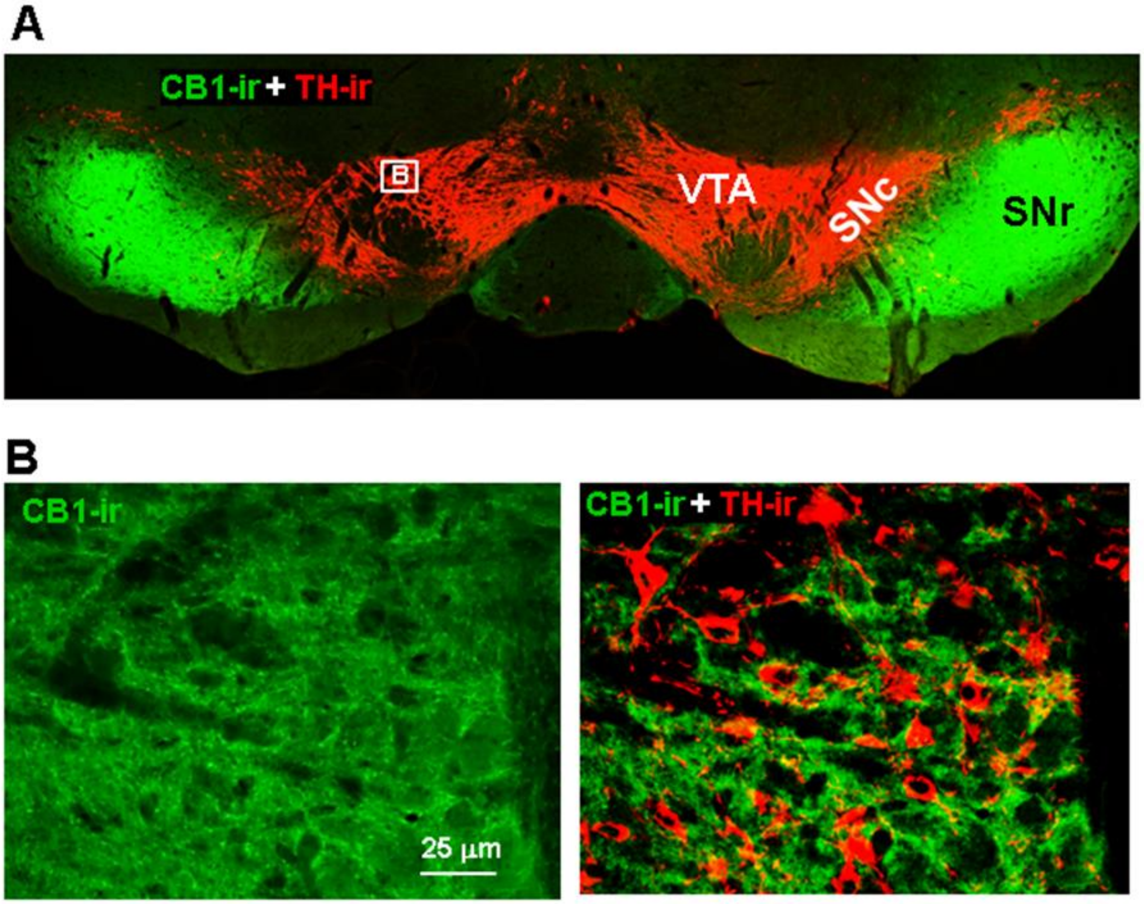

Figure S1 (related to Figs. 1-2): CB1R-immunostaining in the midbrain. **A**: illustrating that CB1Rs are highly expressed in the substantia nigra pars reticulata (SNr), not in the VTA or substantia nigra pars compacta (SNc). **B**: Under high magnification, CB1R-immunostaining was detected mainly in nerve fibers or cell membranes, not in the cell bodies of VTA neurons.

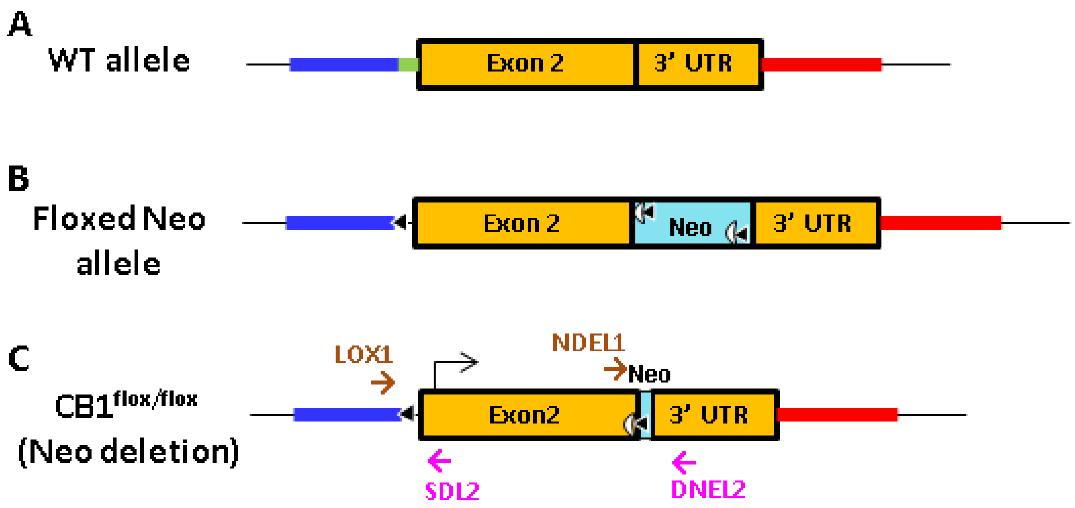

Figure S2 (related to Figs. 1-2) Generation of CB1-floxed (CB1$^{f/f}$) mice. **A**: CB1 allele in wild-type mice. **B**: CB1-floxed-neo allele in the heterozygous CB1$^{flox/wt}$ mice that were used to breed with FLP-Cre mice carrying the germ-line expression of the recombinase flipase to generate CB1$^{flox/flox}$ mice. **C**: CB1 allele in CB1$^{flox/flox}$. In the construct schematic diagrams, blue line represents genomic left arm; red line represents genomic right arm; green line represents splicing region; brown box represents CB1-encoding exon; arrows indicate translation initiation sites for CB1 and Neo genes, black arrow heads indicate loxP sites for Cre recombinase, semicircles indicate FRT sites for Flp recombinase, NDEL1 and NDEL2 indicate screening primers for Neo deletion; Lox1 and SDL2 indicate screening primers for presence of distal loxP site.

Figure S3

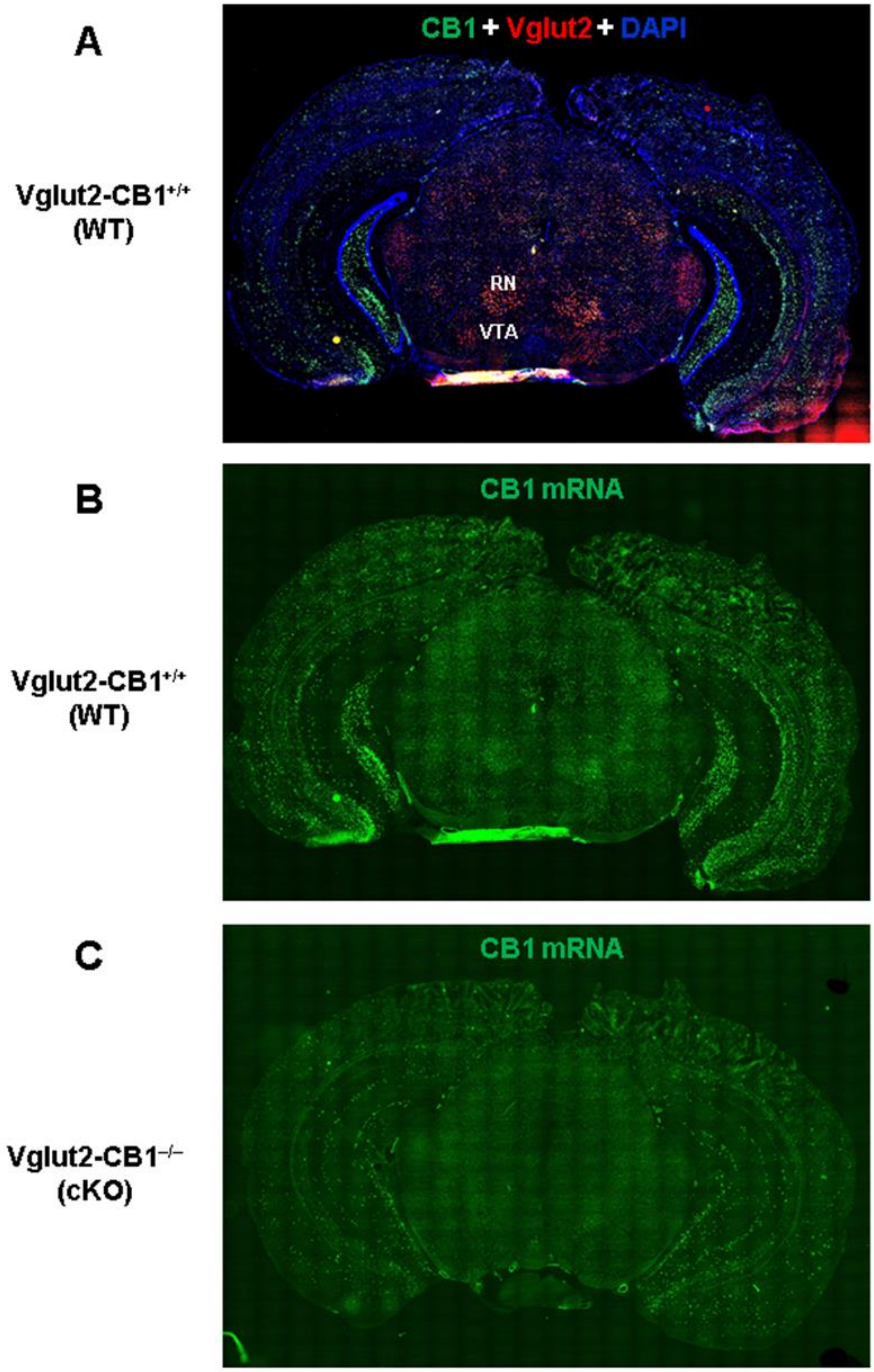
A
CB1 + Vglut2 + DAPI
Vglut2-CB1+/+
(WT)
RN
VTA
B
CB1 mRNA
Vglut2-CB1+/+
(WT)
C
CB1 mRNA
Vglut2-CB1–/–
(cKO)

Figure S3 (related to Figs. 1-2): CB1 mRNA distribution (by RNAscope ISH) in the brain at the midbrain level, illustrating that CB1 mRNA (green) is highly expressed in the hippocampus and cerebral cortex, while VgluT2 mRNA (red) is mainly detected in subcortical regions including the red nucleus (RN) and VTA (**A, B**). However, conditional deletion of CB1Rs in VgluT2+ glutamate neurons decreased CB1 mRNA expression in cerebral cortex and hippocampus, while it abolished CB1 mRNA expression in subcortical regions such as the RN and VTA in VgluT2-CB1$^{-/-}$ mice (**C**).
